# Hidrox^®^ Counteracts Cyclophosphamide-Induced Male Infertility through NRF2 Pathways in a Mouse Model

**DOI:** 10.3390/antiox10050778

**Published:** 2021-05-14

**Authors:** Roberta Fusco, Angela Trovato Salinaro, Rosalba Siracusa, Ramona D’Amico, Daniela Impellizzeri, Maria Scuto, Maria Laura Ontario, Roberto Crea, Marika Cordaro, Salvatore Cuzzocrea, Rosanna Di Paola, Vittorio Calabrese

**Affiliations:** 1 Department of Chemical, Biological, Pharmaceutical and Environmental Sciences, University of Messina, 98166 Messina, Italy; rfusco@unime.it (R.F.); rsiracusa@unime.it (R.S.); rdamico@unime.it (R.D.); dimpellizzeri@unime.it (D.I.); dipaolar@unime.it (R.D.P.); 2 Department of Biomedical and Biotechnological Sciences, University of Catania, 95131 Catania, Italy; trovato@unict.it (A.T.S.); mary-amir@hotmail.it (M.S.); marialaura.ontario@ontariosrl.it (M.L.O.); calabres@unict.it (V.C.); 3 Oliphenol LLC, 26225 Eden Landing Road, Unit C, Hayward, CA 94545, USA; robertocrea48@gmail.com; 4 Department of Biomedical, Dental and Morphological and Functional Imaging, University of Messina, Via Consolare Valeria, 98125 Messina, Italy

**Keywords:** Nrf2, oxidative stress, inflammation, cyclophosphamide

## Abstract

Background: Every year, men use cyclophosphamide to treat various cancers and autoimmune diseases. On the one hand, this chemotherapy often has the beneficial effect of regressing the tumor, but on the other hand, it leads to infertility due to excessive oxidative stress and apoptosis in the testes caused by its metabolite, acrolein. Methods: The objective of this study was to evaluate the beneficial power of a new compound called Hidrox^®^, containing 40–50% hydroxytyrosol, in counteracting the damage related to fertility induced by cyclophosphamide. The study was conducted using a single intraperitoneal injection of cyclophosphamide at a dose of 200 mg/kg b.w, in distilled water at 10 mL/kg b.w. The treatment was administered via the oral administration of Hidrox^®^ at a dose of 50 mg/kg. Results: Our study confirms that the use of cyclophosphamide causes a series of sperm and histological alterations strongly connected with oxidative stress, lipid peroxidation, and apoptosis. Conclusion: Our results demonstrate for the first time that Hidrox^®^ protects testes from CYP-induced alterations by the modulation of physiological antioxidant defenses.

## 1. Introduction

Cancer is one of the most dangerous diseases in the world today. As a result, scientists have looked for ways to prevent it and/or improve patients’ quality of life. More than two-thirds of human cancers are believed to be preventable by a healthy lifestyle, which requires good nutrition [1]. Cyclophosphamide (CYP) (N,N-bis(2-choloroethyl)tetrahydro-2H-1,3,2-oxazaphosphorin-2-amine 2-oxide) is an alkylating agent with an important cytotoxic effect that is commonly used as an anticancer or immunosuppressive treatment [2,3,4]. In particular, lymphoma, multiple myeloma, leukemia, prostate and breast cancer, neuroblastoma, and sarcoma are also treated with it as a method of chemotherapy [5,6]. It is also used to treat nephrotic syndrome, granulomatosis with polyangiitis, and organ transplant rejection, among other conditions [7,8,9].

Despite its wide spectrum of clinical uses, CYP has several adverse effects, such as lack of appetite, vomiting, hair loss, and bladder bleeding. Additionally, an increased potential risk of cancer, miscarriage, allergic reactions, and pulmonary fibrosis are among the most serious side effects [3,4,10]. CYP has been shown to increase the risk of premature menopause in females and infertility in both males and females, with the risk rising with cumulative drug dosage and patient age. Infertility of this kind is normally temporary, but it may also be permanent [5,6]. Through to the cytochrome P450, CYP is metabolized first into two unstable intermediates, 4-hydroxycyclophosphamide and aldophosphamide and following into two stable toxic intermediates: phosphoramide mustard and acrolein [11,12]. Phosphoramide mustard prevents cell division by forming cross-linkages in DNA both between and within DNA strands at guanine N-7 positions. This is irreversible and leads to cell death [13].

Conversely, acrolein—a reactive aldehyde—possesses the ability to generate toxic ROS and subsequently effect surrounding tissue [14,15]. ROS has multiple impacts, including inhibition of a variety of enzymes, membrane and DNA damage, and lipid peroxidation, which contribute to infertility [15,16]. Acrolein exposure can also occur through a variety of other sources, including cigarette smoke, industrial pollution, and other environmental exposures that are all associated with an enhancement of ROS [15,16,17,18]. Oxidative stress (OS) is a well-known key player in the pathogenesis of idiopathic male infertility [19]. In fact, increased testicular oxidative stress may trigger shifts in the patterns of testicular microvascular blood flow and endocrine signaling, contributing to a rise in germ cell apoptosis and eventual hypo-spermatogenesis, making it a main factor in the etiology of male infertility [20]. OS has been shown to alter epigenetic markers and gene expression in somatic cells and gametes in recent studies. DNA methylation, noncoding RNAs, histone modifications, and chromatin remodeling are all epigenetic pathways that have been shown to affect gene expression [19,21,22,23]. The human body has a very effective antioxidant network to defend against OS, involving primary enzymes such as superoxide dismutase (SOD), glutathione (GSH), and catalase (CAT) as well as inducible phase II detoxifying enzymes such as heme oxygenase-1 (HO-1) and NAD(P)H dehydrogenase (quinone) 1 (NQO1) through the activation of nuclear transcription factor-erythroid 2 related factor (Nrf2) [24,25]. Under physiological conditions, Nrf2 is located in the cytoplasm bound to its negative regulator, Kelch-like ECH-associating protein 1 (Keap1). However, upon exposure to ROS, Nrf2 is released from the Keap1–Nrf2 complex and translocates to the nucleus, where it sequentially binds to the antioxidant response element (ARE), a regulatory enhancer region within gene promoters. This binding induces the production of several detoxifying and antioxidant enzyme genes, which protect cells from oxidative stress and a broad range of other toxins [26,27]. When production of ROS exceeds the scavenging capacity of the antioxidant response system, extensive lipid peroxidation and protein oxidation occurs, causing incorrect cellular functioning [28]. For example, it has also been suggested that Nrf2 expression is required for normal spermatogenesis and sperm-specific functions such as motility, and that it can be reduced when spermatozoa are exposed to high levels of ROS [29,30,31,32].

In recent years, the concept that food can act as medicine is getting more and more support from the scientific community; many illnesses can be prevented or limited with diets abundant in plant foods and low in processed products [24,33,34,35,36,37,38,39,40,41,42]. For this reason, it is mandatory to continue research to find a possibly natural compound that can protect against CYP-induced oxidative stress while still reducing chemotherapy-related toxicity. The Mediterranean Diet (MD) is the best example of a diet rich in antioxidants owing to its high fruit and vegetable content. The beneficial effects of the MD are generally due to the action of nutrients found in the foods eaten, but the overall dietary pattern may also improve the body’s endogenous defenses by mechanisms that are still unknown [43]. Vitamin antioxidants and polyphenols in the diet have been extensively studied as an exogenous defense mechanism against oxidative stress and systemic inflammation [44,45,46,47,48]. The likelihood of heart failure, stroke, coronary artery disease, and cancer are significantly decreased after the introduction of antioxidants into diet. Vitamins and polyphenols, which are found primarily but not exclusively in fruits, vegetables, whole grains, nuts, and oil, are abundant in the MD [49].

Olive oil (OO) is, without a doubt, the most common and well-known compound of the MD. This oil comes from the fruit of the olive tree (*Olea europea L.)*. Due to their high hydroxytyrosol (HT) contents, all forms of OO are interesting in terms of their aroma and palatability. However, only those obtained by pressure are interesting clinically due to their high HT content [42]. HT is one of the most common phytochemicals presented in oil and table olives [50]. Numerous reports confirm its value, highlighting HT’s strong antioxidant capacity and potential to destroy free radicals [50,51,52,53]. Different studies demonstrate its cardioprotective, anticancer, neuroprotective, and antimicrobial effects [50,54,55,56,57,58,59,60,61,62,63,64,65,66,67]. Additionally, HT interacts with proteins involved in cell cycle regulation and gene expression and possesses anti-inflammatory, antiatherogenic, and antithrombotic properties [58,59,60,61,62]. Due to the biological HT properties and its safety profile—with no adverse effects in a mouse model, even at very high doses—and with this background in our mind, the focus of our study was to investigate whether the aqueous extract of the olive pulp containing 40–50% of HT, known as Hidrox^®^ (HD), was within the grade required to limit the alteration of testicular tissue, oxidative stress, and CYP-induced apoptosis [63].

## 2. Materials and Methods

### 2.1. Animals

CD1 male mice (8-weeks-old, 18–24 g) were acquired from Envigo (Milan, Italy), placed in a controlled environment and provided with standard rodent chow (Teklad standard diet acquire from Envigo) and water available *ad libitum*. They were housed with five mice/cage and maintained in a 12:12 h light–dark cycle at 21 ± 1 °C and 50 ± 5% humidity. The University of Messina Review Board for animal care (OPBA) approved the study (protocol number n° 211/2021-PR dated 3/16/2021). In addition, the experiments on mice complied with U.S. regulations (Animal Welfare Insurance No. A5594-01, Department of Health and Human Services, Washington, DC, USA), Europe (OJ of ECL 358/1 12/18/1986), and Italy (DM 116192).

### 2.2. Experimental Design and Groups

To reproduce testicular damage, we used a consolidated mice model with a single intraperitoneal (i.p.) injection of CYP (200 mg/kg b.w, i.p.) in distilled water (10 mL/kg b.w) [24,41,64]. For the polyphenol treatment, mice received an oral administration of HD at a dose of 50 mg/kg. HD was kindly provided by Oliphenol LLC. (Hayward, CA, USA). HD is a freeze-dried powder prepared from the aqueous portion of olives [65]. A total of 12% of the HD extract is made up of polyphenols. Among these, the most abundant in HD is hydroxytyrosol, 40–50%, while 5–10% is oleuropein, 0.3% is tyrosol, and about 20% is oleuropein aglycone and gallic acid [66,67].

In particular, after the CYP injection, we randomly divided the animals into three different groups:

(1) Sham: animals were subjected to injections of saline and treated by oral gavage with Hidrox dissolved in saline.
(2) CYP: animals were subjected to CYP injections as described above and treated by oral gavage with saline.
(3) CYP + Hidrox (50 mg/kg): animals were subjected to CYP injections as described above and treated by oral gavage 1 h after CYP-injection and for the following 5 days with Hidrox dissolved in saline.

At the end of the experiment, animals were anesthetized with ketamine (2.6 mg/kg) and xylazine (0.16 mg/kg) and subsequently beheaded. Testis and serum were collected for histology and biochemical analysis. The dose of HD was calculated based on previously published works [53,68,69,70].

### 2.3. Evaluation of Sperm

To obtain the sperm, the entire epididymis from the mouse was minced in a sperm-washing medium and incubated for 30 min at room temperature. The sperm parameters were evaluated as previously described in other work [64,71]. The sperm characteristics were determined according to the guidelines of the World Health Organization (WHO) in the WHO Laboratory Manual for the Examination and Processing of Human Semen, 2010. These are considered valid and are also used in the case of animal sperm.

### 2.4. Western Blot Analysis of Cytosolic and Nuclear Extracts

Cytosolic and nuclear extracts were prepared as previously described [72,73]. The following primary antibodies were used: anti-NRF-2 (1:500, Santa Cruz Biotechnology, Heidelberg, Germany, #sc-365949), anti-Heme Oxigenase 1 (HO-1; 1:500, Santa Cruz Biotechnology, Heidelberg, Germany, #sc-136960), anti-Bax (1:500, Santa Cruz Biotechnology, #sc7480), and anti-Bcl-2 (1:500, Santa Cruz Biotechnology, #sc7382) in 1× PBS, 5% w/v nonfat dried milk, and 0.1% Tween-20 at 4 °C overnight. To ensure that blots were loaded with equal amounts of proteins, they were also probed with antibodies against the β-actin protein for cytosolic fraction (1:500; Santa Cruz Biotechnology, Heidelberg, Germany,) or lamin A/C for nuclear fraction (1:500 Sigma-Aldrich Corp., Milan, Italy). Signals were examined with an enhanced chemiluminescence (ECL) detection system reagent according to the manufacturer’s instructions (Thermo, Monza, Italy). The relative expression of the protein bands was quantified by densitometry with BIORAD ChemiDocTM XRS^+^ software and standardized to the β-actin and lamin A/C levels.

### 2.5. Testosterone Assay

For testosterone assessment, blood samples were collected from the heart. The serum was separated from blood with 15 min of centrifugation at 3000× *g* and stored at −20 °C for testosterone analysis. Serum testosterone levels were in accordance with the manufacturer’s instructions (Mouse Testosterone ELISA Kit, Bioassay, San Francisco, CA, USA, Cat. #E0260MO). The amount of testosterone is expressed as nmol/L. All samples were analyzed in duplicate.

### 2.6. Histopathological Evaluation

Testis were dehydrated, embedded in paraffin, and stained in hematoxylin/eosin (H/E), as previously described [72]. Investigation of testicular damage was assessed considering Johnsen’s score (JS) system from 0 (No seminiferous epithelial cells; tubular sclerosis) to 10 (Full spermatogenesis) [64,74]. Sections from each mouse were observed using a Leica DM6 microscope (Leica Microsystems SpA, Milan, Italy) and scored in a blinded fashion.

### 2.7. Evaluation of Tissue Lipid Peroxidation

The lipid peroxidation levels in terms of thiobarbituric acid reactive substance (TBARS) formation was assessed following the method of Esterbauer and Cheeseman. Briefly, tissue homogenates (containing 1 mg of protein) with the buffer were incubated for 1 h at room temperature. After this, they were treated with trichloroacetic acid (1 mL, 20%) and thiobarbituric acid (2 mL, 0.67%), then kept in a boiling water bath for 30 min. After cooling, the precipitate was removed by centrifugation at 1000× *g* for 15 min.

The quantity of TBARS formed was measured by taking the absorbance of the supernatant at 532 nm [75].

### 2.8. Assessment of Tissue Antioxidant Activity

SOD and CAT activity and GSH concentration were examined as previously described in other works [24,76,77,78]. Briefly, SOD activity was assessed according to the change in absorbance of samples at 560 nm. CAT was measured as the number of enzymes, which reduces by 1 mmol of H_2_O_2_/min. GSH activity was assessed with an absorbance at 417 nm and calculated using a standard curve.

### 2.9. Materials

Unless otherwise stated, all the compounds were purchased from Sigma-Aldrich.

### 2.10. Statistical Evaluation

All the values are expressed as the mean ± standard error of the mean (S.E.M.) of N observations. For in vivo studies, N represents the number of animals used. The results were analyzed by one-way ANOVA followed by a Bonferroni post-hoc test for multiple comparisons. A *p*-value less than 0.05 was considered significant.

## 3. Results

### 3.1. Impact of HD Administration on Sperm Parameters and Testosterone Levels after CYP

**Figure 1:**
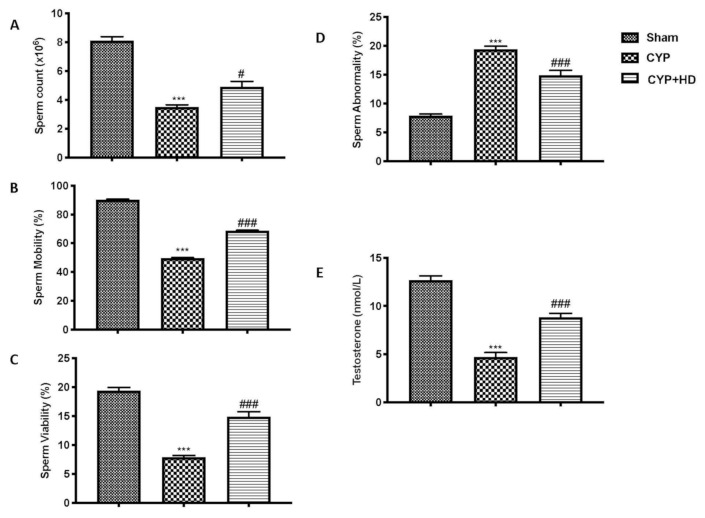
Impact of HD administration on sperm parameters and testosterone levels after CYP administration. Analysis of sperm parameters: sperm count (**A**), sperm motility (**B**), sperm viability (**C**), sperm abnormality (**D**), and testosterone serum level (**E**). *** *p* < 0.001 vs. sham; # *p* < 0.05 vs. CYP; ### *p* < 0.001 vs. CYP.

As we expected, CYP administration causes a considerable alteration in sperm parameters. In particular, we observed a decrease in sperm count (Figure 1A), motility (Figure 1A,B), and viability (Figure 1A,C). These alterations are reflected in the increase in the abnormality of the CYP-induced sperm (Figure 1A,D). Additionally, harm to the testes was shown by the significant difference in testosterone levels between the CYP-treated and other groups (Figure 1A,E). HD administration was capable of restoring—at almost all levels of the control groups—all the sperm parameters considered as well as the levels of testosterone. They indicate that these abnormalities may result directly from DNA damage or at specific levels of differentiation of spermatozoa.

### 3.2. Influence of HD Administration on Histological Testis Architecture

**Figure 2:**
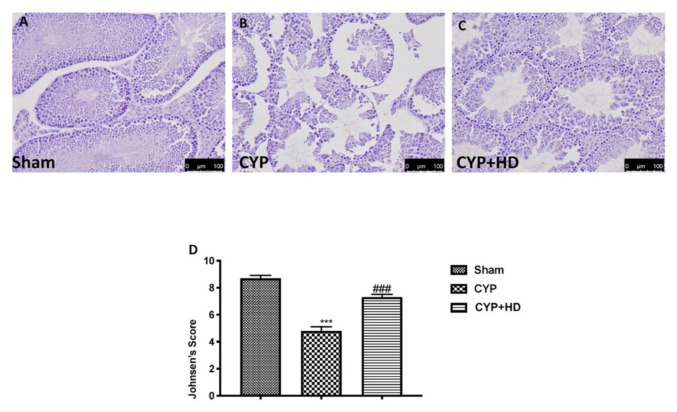
Impact of HD administration on histological testis architecture. Histological photographs of testicular tissue of sham (**A**), CYP (**B**), and CYP + HD (**C**). Graphical representation of Johnsen’s Score (**D**). *** *p* < 0.001 vs. sham; ### *p* < 0.001 vs. CYP.

The data obtained from the sperm analysis prompted us to continue the study by investigating the morphological alterations that occur after treatment with CYP. The sham group (Figure 2A, and Johnsen’s score D) shows a normal architecture of the testis with well-defined seminiferous tubules at different stages of spermatogonial cells. Additionally, a lumen occupied with spermatozoa and interstitial cells are visible. Moreover, Sertoli cells are visible in different sections as well as Leydig cells, having prominent cytoplasm and well-defined nuclei. Testicular tissues after CYP appear with a smaller number of spermatozoa in lumen (reduction of the number of spermatozoa) and disorganized spermatogenic cells with a reduced number of spermatids. Additionally, the epithelial wall of seminiferous tubules was observed to be damaged and disrupted, and an increase in interstitial edema was shown (Figure 2B, and Johnsen’s score D). After HD administration at a dose of 50 mg/kg, testicular tissue appears restored with a normal number of spermatozoa, reduction in edema, and luminal disruption (Figure 2C, and Johnsen’s score D).

### 3.3. HD Ameliorate Oxidative Endogenous Defence after CYP

**Figure 3:**
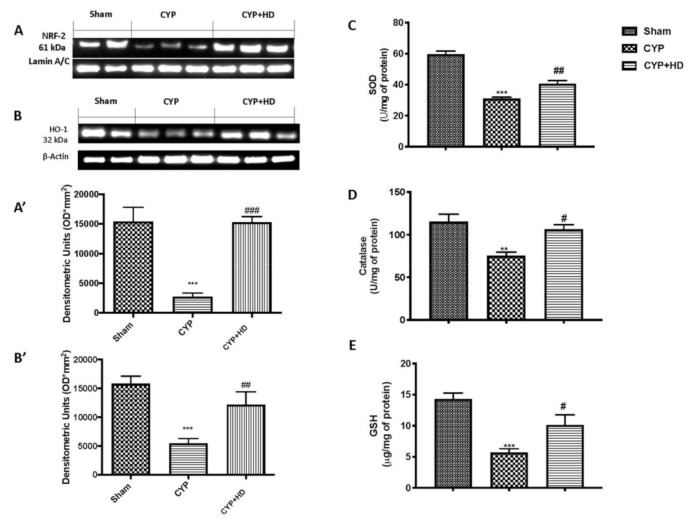
HD stimulates oxidative endogenous defense. Western blots and quantification of testicular tissue for Nrf2 (**A**,**A’**) and HO-1 (**B**,**B’**), respectively. Analysis of SOD (**C**), catalase (**D**), and GSH (**E**) levels after CYP induction. *** *p* < 0.001 vs. sham; ** *p* < 0.01 vs. sham; # *p* < 0.05 vs. CYP; ## *p* < 0.01 vs. CYP; ### *p* < 0.001 vs. CYP.

Our results, obtained by western blot analysis, showed a significant decrease in Nrf2 expression compared to sham mice after CYP injection (Figure 3A,A’). Additionally, considering that HO-1 is an Nrf2-regulated gene that plays a critical role in maintaining antioxidant/oxidant homeostasis, we also investigated its expression by western blotting. In line with Nrf2, HO-1 (Figure 3B,B’) also decreases after CYP. These decreases were almost completely restored after the administration of HD at the dose of 50 mg/kg.

Additionally, we investigated these markers by ELISA kit and found that HD at the dose of 50 mg/kg was of a suitable grade to significantly counteract CYP-induced decrease, almost completely restoring SOD (Figure 3C) and catalase (Figure 3D) expression as well as GSH (Figure 3E) activity at a physiological level.

### 3.4. HD Limits CYP-Induced Apoptosis

**Figure 4:**
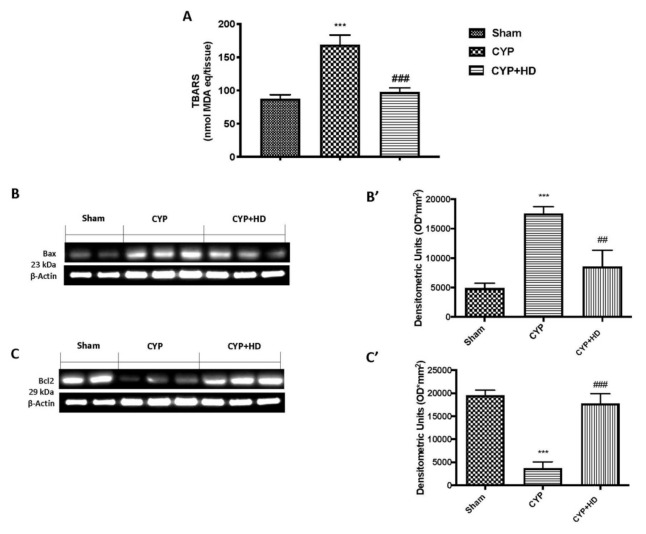
HD limits lipid peroxidation and CYP-induced apoptosis according to TBARS analysis (**A**), western blot and quantification of testicular tissue from Bax (**B**,**B’**), and BCL-2 (**C**,**C’**), respectively. *** *p* < 0.001 vs. sham; ## *p* < 0.01 vs. CYP; ### *p* < 0.001 vs. CYP.

Under oxidative stress, lipid peroxidation is a degenerative process that affects unsaturated membrane lipids [79]. In accordance with the previous result, we found a significant increase in TBARS in the CYP-induced group compared to control groups (Figure 4A); however, after HD administration at 50 mg/kg, we saw a significant decrease.

It is well known that apoptosis is closely connected with the oxidative stress. By western blot analysis, we investigated the expression of Bax and Bcl-2 and found that in the CYP-induced testicular damage, we observed an increase in Bax (Figure 4B,B’) and a decrease in Bcl-2 (Figure 4C,C’) expressions compared to control groups. Conversely, HD was of a suitable grade to significantly decrease Bax and increase Bcl-2 expression.

## 4. Discussion

CYP is a cytotoxic bifunctional alkylating agent that belongs to the nitrogen mustard family of drugs and is used to treat several different tumors as well as during organ transplant rejection or autoimmune diseases [80,81,82,83]. However, despite its broad range of therapeutic applications, it causes extreme cytotoxicity in humans and animals, particularly in the reproductive organs, urinary tract, and liver [40,84,85,86,87,88,89,90,91,92]. CYP is quickly metabolized in aldophosphamide mustard and acrolein, which interfere with cellular DNA synthesis [93]. It is the latter, acrolein, that causes the important oxidative stress and toxicities that destroy the cell’s lipids, proteins, and DNA [94]. Due to its unintended toxicities in normal cells, CYP’s therapeutic effectiveness is restricted [95]. As a result, it is important to avoid CYP-induced DNA disruption in normal cells in clinical settings.

The conviction that “diet is the best medicine” applies to the valuing of food ingredients, especially health-promoting bioactive compounds [96]. The Mediterranean diet trend is described by a high intake of fruits, vegetables, and fish and a lower intake of meats and full-fat dairy products. It also involves regular use of olive oil as the primary fat in culinary practices [53,97,98]. Several studies have been conducted to determine which components of this balanced diet are responsible for these beneficial effects, and the majority of them have identified HT as one of these components [99,100,101].

HT possesses a broad spectrum of actions ranging from antimicrobial, antioxidative, and antiatherosclerotic to anti-inflammatory effects [50]. HD is an aqueous extract of olive oil that contains 40–50% of HT. All the essential elements are preserved in their natural matrix in this compound due to a special manufacturing technique. In addition, many in vitro and in vivo experiments have shown that HD has higher antioxidant and anti-inflammatory properties [67]. Here, using a consolidated model of CYP-induced reproductive impairment in mice, we have shown for the first time that the administration of HD could have a beneficial effect. It is well known that CYP causes temporary interference of normal male reproductive system and testosterone levels [102]. In our study, HD restored not only numbers and vitality of sperm but was also capable of increasing testosterone levels after CYP treatment. The decrease in sperm viability was probably correlated with a series of histological alterations. In fact, CYP causes a reduction in the size and number of seminiferous tubules, spermatogonia degeneration and vacuolation, reduced spermatocytes and germ cells, irregular seminiferous tubules, reduced seminiferous epithelial layers, significant maturation arrest, and perivascular fibrosis [81,103,104]. The histological structure of CYP-treated mice showed reduction in germinal cells, atrophy in tubules, vacuolization in Sertoli cells, interstitial edema and congestion, and reduced diameters of seminiferous tubules and Johnsen’s Testicular Score [64]. HD at the dose of 50 mg/kg was able to limit CYP-induced histological alteration. It has been demonstrated that spermatocytes and spermatids can produce low levels of ROS, which are required for many physiological processes such as capacitation, hyperactivation, and sperm–oocyte fusion [105]. Spermatozoa are particularly vulnerable to damage caused by excessive ROS because their plasma membranes contain large amounts of polyunsaturated fatty acids and their cytoplasm contains low concentrations of scavenging enzymes. Spermatogenesis is a complex mechanism in which male germ cells proliferate and develop from diploid spermatogonia to mature haploid spermatozoa via meiosis [106]. The mechanism includes complex interactions between germ cells in development and the Sertoli cells that sustain them [107]. The gonadal tissue is particularly susceptible to overexpression of ROS due to its abundance of highly unsaturated fatty acids, high rates of cell division, and variety of testis enzymes. To combat this risk, testes have evolved a complex antioxidant system that includes both enzymes and free radical scavengers [107,108,109]. Endogenous antioxidant enzymes, such as SOD, GSH, and CAT or Nrf2 stimulation, are primarily responsible for removing excessive ROS [105]. An increasing amount of data suggests that HT stimulates the phase 2 reaction, resulting in increased expression of Nrf2 [110,111]. In vitro and in vivo data suggest that Nrf2 is involved in the regulation of oxidative and apoptotic responses in a variety of cell types and diseases [112,113]. The levels of Nrf2 in the testes were found to be lower when CYP was used. Furthermore, CYP therapy disrupted Nrf2 downstream pathway molecules such as HO-1 and SOD and the physiological antioxidant response [114]. It is noteworthy that HD greatly increased the amount of Nrf2 as well as HO-1 and significantly enhanced physiological antioxidant response through the increase in catalase and GSH activity. In an autocatalytic process, ROS can attack the unsaturated bonds of membrane lipids, resulting in the formation of peroxides, alcohol, and lipidic aldehydes as by-products [115,116,117]. As a result, an increase in free radicals in cells can cause lipid peroxidation in cell membranes due to oxidative breakdown of polyunsaturated fatty acids [64,118,119]. HD oral administration at the dose of 50 mg/kg was of a suitable grade to counteract lipid peroxidation, as demonstrated by the decrease in TBARS. Lipid peroxidation products interact with membrane receptors and transcription factors/repressors to activate apoptosis signaling. It can cause both intrinsic and extrinsic apoptotic signaling pathways to be activated. Cardiolipin peroxidation, a mitochondrion-specific inner membrane phospholipid, and subsequent products of lipid peroxidation formation have been linked to the activation of intrinsic apoptosis [120,121,122]. In our work, we noticed a significant increase in Bax expression and a significant decrease in Bcl-2. HD supplementation at the dose of 50 mg/kg was capable of nearly completely restoring both expressions at the physiological level.

## 5. Conclusions

The imbalance in generation and elimination of ROS is often associated with various pathologies that lead, as a final consequence, to cell death. The cell is shielded from oxidative damage by the general endogenous antioxidant mechanism, which consists of a series of enzymatic antioxidants that are able to limit ROS production. The reproductive system physiologically requires ROS for reproduction, but the risk represented by an uncontrolled generation of these must be reduced by stimulating physiologically antioxidative systems when required, perhaps by a diet rich in natural antioxidant compounds. Our study demonstrates for the first time that the oral administration of HD at a dose of 50 mg/kg could be helpful in reducing excess oxidative stress and preventing sperm damage, lipid peroxidation, and apoptosis in the testes following CYP injection.

## Data Availability

The data presented in this study are available on request from the corresponding author.
